# Step-Free Coating Technique for Four-Quadrant Wind Imaging Interferometer

**DOI:** 10.3390/s25175385

**Published:** 2025-09-01

**Authors:** Tingyu Yan, Luhan Huang, Yanqiang Wang, Chunmin Zhang

**Affiliations:** 1 Key Laboratory of Atmospheric Sounding, College of Electronic Engineering, Chengdu University of Information Technology, Chengdu 610225, China; 3240308010@stu.cuit.edu.cn; 2 Shaanxi Province Key Laboratory of Thin Films Technology and Optical Test, School of Opto-electronical Engineering, Xi’an Technological University, Xi’an 710021, China; wangyanqiang@xatu.edu.cn; 3 School of Physics, Xi’an Jiaotong University, Xi’an 710049, China; zcm@xjtu.edu.cn; 4 MOE Key Laboratory for Nonequilibrium Synthesis and Modulation of Condensed Matter, Xi’an Jiaotong University, Xi’an 710049, China

**Keywords:** wind imaging interferometer, step-free coating, phase steps

## Abstract

The four-quadrant wind imaging interferometer achieves wind field measurement by acquiring interferograms of the target scene in four distinct phase states while maintaining identical radiation intensity. This unique operational principle endows the system with notable advantages, including low sensitivity to temporal variations of the light source and exceptional compactness in instrument design. This paper proposes a step-free, four-quadrant, stepped-phase coating technique, which is applicable to an all-solid-state wind imaging interferometer scheme while offering advantages such as high reflectivity and improved stepped-phase precision for quadrants of wind imaging interferometers. Beginning with the underlying principles, we provide a detailed analysis of the four-quadrant material selection and coating architecture. This is followed by computer-aided thin-film design and simulation for verification.

## 1. Introduction

Space-based observations indicate that tides, planetary waves, and gravity waves of various scales not only influence atmospheric motions in the mesosphere and lower thermosphere regions but also play a crucial role in driving the large-scale circulation of the middle atmosphere. However, the underlying mechanisms of coupling, energy dissipation, and their interactions remain subjects of ongoing research. Thus, there is a need for high temporal and spatial resolution, as well as high-precision detection of the dynamical processes in the middle atmosphere [1]. The middle and upper atmospheric wind fields constitute crucial parameters in atmospheric dynamics research. Precise measurement of wind profiles significantly enhances our understanding of atmospheric dynamical processes and climatic mechanisms, which can contribute to the optimization of numerical forecasting systems and the improvement of the predictive capabilities of climate models [2]. Advances in remote sensing technology for atmospheric wind fields are driving humanity’s understanding and control of the Earth’s system. The ADM-Aeolus satellite, which carries the world’s first space-based Doppler Wind Lidar (DWL) system, utilizes the Doppler effect to achieve three-dimensional observation of global wind fields. This advancement enhances atmospheric dynamics research and climate model evaluations, filling the gaps left by traditional meteorological observations [3,4]. The Doppler effect, due to its high precision and non-contact wind measurement capabilities, has become the core principle for atmospheric wind field detection.

Satellite-borne remote sensing stands as a pivotal method for global measurements of horizontal wind fields in the middle and upper atmosphere, typically utilizing limb-sounding techniques to acquire altitude-resolved wind profile data. The Wind Imaging Interferometer (WINDII), launched on the U.S. UARS satellite in 1991, was the first successful spaceborne wide-field Michelson wind imaging interferometer. It measures Doppler frequency shifts by analyzing the phase shifts of interference patterns generated from a single airglow line [5,6]. Its prototype was the Wide-Angle Michelson Doppler Imaging Interferometer (WAMDII), developed by Shepherd et al. at York University [7]. Subsequently, a diverse array of passive wind imaging interferometers has been developed, each operating on distinct foundational principles and tailored to specific atmospheric detection scenarios—ranging from upper atmospheric wind profiling to mesospheric wind field mapping. For instance, the Polarizing Atmospheric Michelson Interferometer (PAMI) developed in 1995 employed a polarization interference principle, where optical path differences were modulated by rotating external polarizers [8]. In 2001, William E. Ward et al. proposed the Waves Michelson Interferometer (WAMI), which includes visible and near-infrared channels. WAMI utilizes segmented interferometers to sample fringes simultaneously along different optical paths, allowing for atmospheric observation from several different airglow bands [9]. In 2006, the Mesospheric Imaging Michelson Interferometer (MIMI) was introduced, featuring an innovative four-quadrant coating configuration for precise mesospheric wind measurements [10]. Additionally, the ground-based Michelson Interferometer for Airglow Dynamics Imaging (MIADI) and the E-Region Wind Interferometer (ERWIN) have been implemented to measure upper atmospheric motions using airglow emissions, all based on the moving-mirror Michelson interferometer [11,12,13]. In 2013, the Birefringent Doppler Wind Imaging Interferometer (BIDWIN) concept was developed in collaboration with industry to create an instrument that is simple to construct and capable of observing upper atmospheric winds using low-intensity airglow emissions [14,15]. Meanwhile, the Michelson Interferometer for Global High-resolution Thermospheric Imaging (MIGHTI) was proposed to measure horizontal wind speed profiles and thermospheric temperatures in the altitudinal range of 90 km to 300 km. It was launched aboard the Ionospheric Connection Explorer (ICON) satellite by NASA in 2019 [16]. In 2023, Chunmin Zhang et al. developed the compact Static Wind Imaging Michelson Interferometer (SWIMI) for measurement of stratospheric wind fields using near-infrared airglow emissions. SWIMI features field widening, an achromatic design, and temperature compensation, making it well-suited for high-precision atmospheric wind measurements [17].

Traditional wind imaging interferometers collect light beams through an objective lens in the Michelson interferometer, which splits the incoming light into two beams. These beams travel through the two arms of the interferometer and are recombined. One arm ends with a fixed mirror, while the other arm ends with a movable mirror (the moving mirror). Displacing the moving mirror creates an optical path difference of approximately one-quarter wavelength, followed by sampling of the interference fringes at four points. To mitigate phase errors caused by variations in source intensity during the moving mirror’s displacement for sequential four-sample acquisition, a quadrant-based interferometer was developed, enabling simultaneous capture of interference patterns at four distinct phase steps [17,18,19].

In 1989, Piotrowski et al. achieved 90° stepped-phase reflection in the wavelength range of 550 nm to 770 nm by depositing alternating high- and low-refractive-index quarter-wave films on a mica plate [20]. It addresses the measurement errors in conventional interferometers caused by temporal variations in scene radiance when generating phase differences by adjusting the position of one mirror. WAMI is capable of detecting both dynamic and compositional characteristics of the atmosphere within the altitude range of 45–180 km. Specifically, this instrument adopts a configuration that combines a quad-partitioned coated mirror with piezoelectric ceramics. Through this integrated design, it achieves simultaneous detection in both the visible-light band and the infrared band [9]. Building on the foundational design and operational principles of WAMI, significant advancements have been made in related phase-stepping mirror technology in recent years. In 2022, Samuel K. Kristoffersen et al. achieved a notable breakthrough by conducting the first laboratory demonstration of a quad-partitioned phase-stepping mirror specifically engineered for wind imaging applications within a field-widened Doppler imaging interferometer [21]. In 2023, the SWIMI prototype employed glass components with achromatic, temperature-compensated, and field-widened properties, thereby achieving effective field widening and thermal stability. Additionally, a four-quadrant reflective mirror was integrated into one arm of the interferometer to enable phase stepping of the interference signal. The ability to measure two-dimensional wind fields with high temporal and spatial resolution was validated through laboratory experiments [17].

However, the currently known four-zone coating technologies for interferometers rely on non-uniform coating systems with steps. In traditional four-zone coating processes, the film system is deposited on the front surface of the glass substrate, where light incidence occurs. The step phase in such systems is entirely regulated by the thickness of the film layer, rendering it highly sensitive to wavelength variations. Given that the coating is applied to the front surface, an inevitable air gap exists between the field-widening glass and the step-phase mirror. This introduces medium transitions during light transmission, thereby leading to optical losses. Furthermore, the film layer being exposed to ambient air imposes stricter requirements on environmental conditions such as temperature, air pressure, and cleanliness. This paper proposes a novel step-free, quad-zone, stepped-phase coating technique designed for film system fabrication on the backside of a glass substrate (compensation glass). It features high reflectivity and precise stepped-phase control while establishing a basis for all-solid-state compensation in static four-zone coated wind imaging interferometers.

## 2. Principle

### 2.1. Principle of the Static Wind Imaging Michelson Interferometer

**Figure 1:**
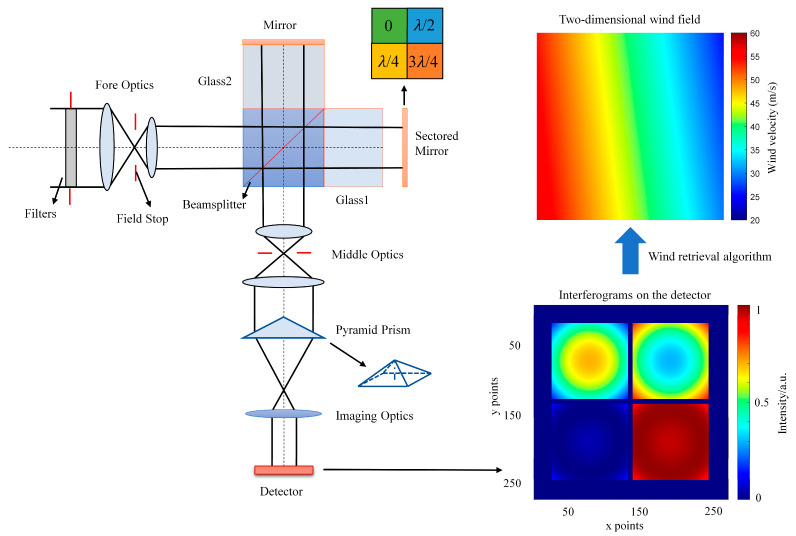
The optical layout of the SWIMI system (**left**), four corresponding interference fringe images on the detector (**lower right**), and the expected two-dimensional wind field across the wind wheel in the experiment (**upper right**) [17].

To elaborate on the technical requirements of four-zone coating—particularly its purpose of introducing precise phase differences for wind field measurement—this section takes the SWIMI instrument as a case study to outline the principle of the optical system of the Michelson Wind Imaging Interferometer and its approach to wind field detection. Figure 1 illustrates the optical layout of the SWIMI system, including its key components, such as the fore-optics system, interferometer, relay optics system, and spectral separation system. Airglow radiation enters the interferometer through the fore-optical system and is split into two beams of equal amplitude by the beam splitter. One beam passes through Glass 1 and the air gap and is then reflected by the sectored mirror, whose adjacent segments increase in optical path by one-quarter wavelength. The other beam passes through Glass 2 and is reflected by a flat mirror. The two beams recombine at the output of the beam splitter, pass through the relay optical system, and enter the spectrographic imaging system. Within the spectrographic system, a pyramid prism divides the beam into four interference beams with different phase shifts. Finally, the four beams converge on the detector to produce four corresponding interference fringe images. The interference intensity values of these images are used to retrieve wind field information based on the four-point detection method [22].

The light source employed in SWIMI is the O2 Δ1 (0,0) emission band at 1.27 μm. Its dayglow is primarily distributed within the vertical altitude range of 40 km to 100 km, with a peak intensity of 500 MR under limb observation conditions. Moreover, the background radiation surrounding this band is relatively weak, which helps enhance the signal-to-noise ratio (SNR). These characteristics make it highly favorable for wind field detection. The interferometer features an effective basic optical path difference (OPD) of 10 cm, a parameter that ensures wind measurement precision of better than 5 m/s when the SNR is approximately 150 [17].

Within the SWIMI system, a four-zone sectored mirror is employed to enable static, simultaneous, and real-time wind field detection. As depicted in Figure 1, however, an air gap exists between Glass1 and the sectored mirror. Consequently, the coating for the sectored mirror is deposited on the glass surface where light incidence occurs. Since the stepped-phase magnitude is regulated by the thickness of SiO_2_, the four zones on the sectored mirror’s surface are uneven.

### 2.2. Theoretical Calculation of Thin Films

**Figure 2:**
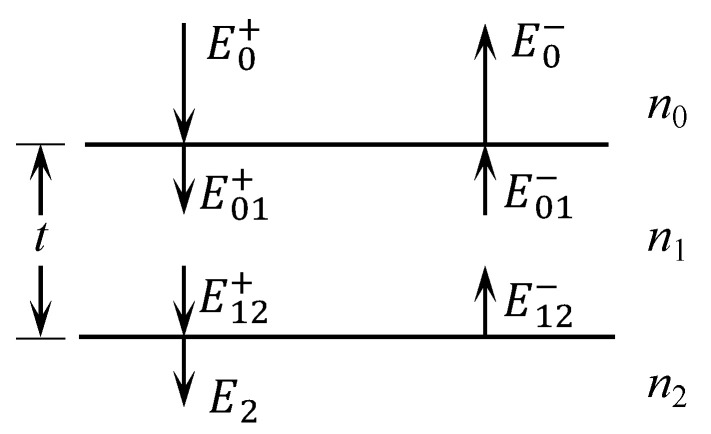
The electric field distribution of a single-layer film.

The study of optical thin-film design is fundamentally an investigation into the propagation characteristics of electromagnetic waves through multilayer dielectric media. For a single-layer thin film, the co-directional electromagnetic waves on both sides of the film can be unified, with the forward and backward directions conventionally denoted by the positive (+) and negative (−) signs, respectively. In Figure 2, the superscript denotes the direction of propagation of the electromagnetic wave, while the subscript indicates the information of the medium.

According to the boundary conditions, the tangential components of the electric field intensity (E) and magnetic field intensity (H) are continuous on both sides of the interface of the optical film, leading to the conclusion that [23,24]

(1) $$\begin{array}{l} E_{0}=E_{0}^{+}+E_{0}^{-}=E_{01}^{+}+E_{01}^{-}\\ H_{0}=H_{0}^{+}+H_{0}^{-}=\eta_{1}E_{01}^{+}-\eta_{1}E_{01}^{-} \end{array},$$

where η is defined as the ratio of the tangential component of magnetic-field intensity to the tangential component of electric-field intensity, which is introduced as the effective admittance. By introducing the phase factor of the wave, we obtain

(2) $$\begin{array}{l} E_{12}^{+}=E_{01}^{+}e^{-i\delta_{1}}\\ \;E_{12}^{-}=E_{01}^{-}e^{i\delta_{1}} \end{array},$$

where $\delta_{1}$ is expressed as

(3) $$\delta_{1}=\frac{2\pi }{\lambda }n_{1}t\cos\varphi_{1},$$

where $\varphi_{1}$ is the light incidence angle and t is the film thickness; thus, the expressions for $E_{0}$ and $H_{0}$ can be written in matrix form as

(4) $$\left[\begin{array}{c} E_{0}\\ H_{0} \end{array}\right]=\left[\begin{array}{cc} e^{i\delta_{1}} & e^{-i\delta_{1}}\\ \eta_{1}e^{i\delta_{1}} & -\eta_{1}e^{-i\delta_{1}} \end{array}\right]\left[\begin{array}{c} E_{12}^{+}\\ E_{12}^{-} \end{array}\right]\;\mathrm{and}$$

(5) $$\left[\begin{array}{c} E_{12}^{+}\\ E_{12}^{-} \end{array}\right]=\left[\begin{array}{cc} \frac{1}{2} & \frac{1}{2\eta_{1}}\\ \frac{1}{2} & -\frac{1}{2\eta_{1}} \end{array}\right]\left[\begin{array}{c} E_{2}\\ H_{2} \end{array}\right].$$

By combining Equations (4) and (5), the following expression can be derived:

(6) $$\left[\begin{array}{c} E_{0}\\ H_{0} \end{array}\right]=\left[\begin{array}{cc} \cos\delta_{1} & \frac{i\sin\delta_{1}}{\eta_{1}}\\ i\eta_{1}\sin\delta_{1} & \cos\delta_{1} \end{array}\right]\left[\begin{array}{c} E_{2}\\ H_{2} \end{array}\right]$$

Through successive linear transformations, the matrix equation for multilayer thin-film structures can be derived. In this equation, the square matrix represents the characteristic matrix of the thin film obtained from the transformation of the multilayer thin-film matrix equation [23,24]:

(7) $$\left[\begin{array}{c} B\\ C \end{array}\right]=\left\{\prod_{\tau =1}^{q}\left[\begin{array}{cc} \cos\delta_{\tau } & \frac{i\sin\delta_{\tau }}{\eta_{\tau }}\\ i\eta_{\tau }\sin\delta_{\tau } & \cos\delta_{\tau } \end{array}\right]\right\}\left[\begin{array}{c} 1\\ \eta_{q+1} \end{array}\right]=M\left[\begin{array}{c} 1\\ \eta_{s} \end{array}\right]$$

where ${\left[\begin{array}{cc} B & C \end{array}\right]}^{T}=\frac{1}{E_{q+1}}{\left[\begin{array}{cc} E_{0} & H_{0} \end{array}\right]}^{T}$ denotes the characteristic matrix of the combined thin-film and substrate system. Based on the aforementioned theoretical framework, computer-aided design and analysis of multilayer coatings can be effectively implemented. Once the material composition and thickness of each layer are determined, computational methods allow for the rapid derivation of optical thin-film parameters (such as reflectance and reflection phase), as well as comprehensive performance evaluation. Consequently, the core challenge in designing a four-quadrant, stepped-phase thin-film system lies in the strategic selection of layer architectures, material combinations, and thickness optimization strategies.

### 2.3. Selection of Thin-Film Layer Materials

The core functionality of the four-quadrant, stepped-phase reflective mirror relies on its reflective properties, which can be achieved through various types of reflective coatings, including metallic coatings, all-dielectric coatings, and metal–dielectric coatings. Metallic coatings offer broad spectral response, high reflectivity, and minimal polarization effects but are prone to oxidation, making them suitable for general-purpose mirrors with wideband requirements. All-dielectric coatings, on the other hand, are characterized by extremely high reflectivity, with absorption losses close to zero, often achieving reflectivity over 99%. However, they have a narrow operational bandwidth and exhibit strong polarization effects, typically used in applications requiring high reflectivity and limited bandwidth. Hybrid metal–dielectric coatings combine the advantages of both metallic coatings and all-dielectric coatings, achieving broad operational bandwidth while selectively enhancing reflectivity at specific target wavelengths.

Based on the operational bandwidth and application scenarios of the four-quadrant, stepped-phase reflective mirror, it is necessary to select optical materials with both high and low refractive indices for the design of an anti-reflective coating. Since the mirror operates in the near-infrared band at 1.27 μm, materials with good near-infrared transmittance must be utilized. The four zones should preferably employ identical materials to ensure similar reflectance and phase-angle variation characteristics. Reducing the number of material types can enhance chemical stability and lower costs, thereby achieving a better balance among processing difficulty, performance, and overall system stability. Materials with strong adhesion properties are essential. Protective coatings may be incorporated to further improve the durability of the thin-film system.

In line with the above requirements, silver (Ag) is selected as the reflective substrate material for this sectored mirror-coating technique. Compared to aluminum, silver offers superior stability, while it is more cost-effective to process than gold. Magnesium fluoride (MgF_2_), an optical material with a relatively low refractive index of approximately 1.38, boasts the highest mechanical strength among low-index halide material, making it an ideal low-refractive-index option for the four-quadrant, stepped-phase reflective mirror. Titanium dioxide (TiO_2_), with a high refractive index of around 2.2, maintains optical transparency across the visible to near-infrared spectral range. Aluminum oxide (Al_2_O_3_) is employed as the protective-layer material; it exhibits strong adhesion to silver, demonstrates robust stability, and effectively shields the silver reflective substrate from oxidation and abrasion.

## 3. Methods

### 3.1. Design of the Thin-Film Structure

The main purpose of the design is to address the application requirements of the four-quadrant, stepped-phase reflective mirror in the near-infrared band (1.27 μm). In the current coating-system design for SWIMI, phase stepping across the four sectors is achieved through variations in coating thickness—meaning that differing film-layer thicknesses result in distinct optical path lengths. However, this design comes with certain drawbacks. For one, the optical path length, modulated by thickness, is highly sensitive to wavelength changes. For another, because the surface of the four-quadrant, stepped-phase mirror is coated, an air gap must be maintained between the field-widening glass and the mirror, which compromises the stability of the system as a whole. By optimizing the coating-system design and material selection, it aims to resolve issues such as surface steps and additional air optical path lengths caused by differences in coating thickness in traditional designs. Meanwhile, it ensures that the four quadrants have consistent reflectance and phase-angle variation characteristics; enhances the chemical stability, mechanical strength, and durability of the coating system; and achieves high-performance optical performance of the mirror within the operating bandwidth while reducing processing difficulty and costs. Ultimately, it meets the needs for precise phase control and stable reflection in specific application scenarios.

**Figure 3:**
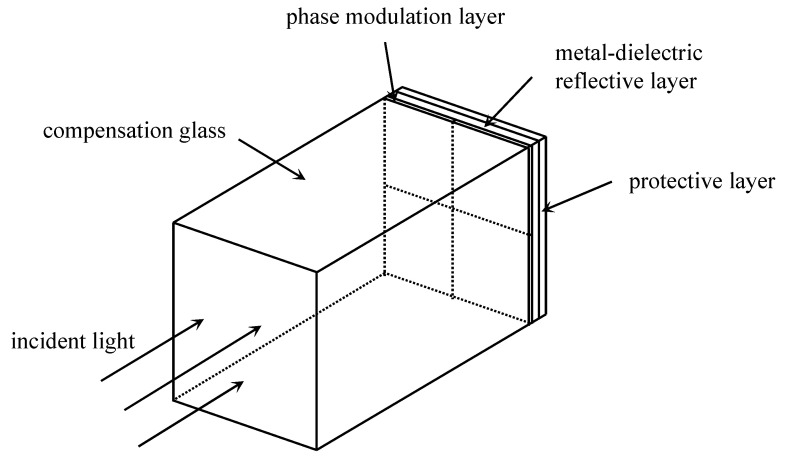
Schematic diagram of the step-free coating structure for a four-quadrant, stepped-phase mirror.

In traditional designs of four-quadrant, stepped-phase reflective mirrors, variations in coating thickness create surface steps, resulting in an uneven surface and introducing extra air optical path lengths. To resolve this issue, the present design adopts a coated backside configuration: the four-quadrant, stepped-phase mirror coating system is deposited on the light exit surface of a glass substrate (referred to as compensation glass). This configuration utilizes the inherent flatness of the glass substrate to eliminate the stepped discontinuities present in the four-quadrant coating system. To reduce the difficulty of the coating process for the quadripartite stepped-phase reflective mirror, it is advisable to carry out the simultaneous evaporation of all four sections during multilayer repetitive coatings. The layer at the forefront of Figure 3 (adjacent to the glass surface) is the stepped-phase modulation layer, followed sequentially by the metal–dielectric reflective layer and the protective layer. The first stepped-phase modulation layer requires separate deposition for each of the four zones using a masking technique. In contrast, the subsequent metal–dielectric reflective layers and protective layers are identical across all four zones and can therefore be deposited simultaneously.

### 3.2. Coating System Design

Computer-aided design and analysis are implemented based on the aforementioned research discussions. TFCalc_v3.5.6 optical thin-film design software was used to design and analyze a four-quadrant, stepped-phase reflective coating. A material library is established on the computer, and the relevant environmental coating parameters are set as shown in Table 1.

The first layer of the four-quadrant, stepped-phase reflector employs MgF_2_ as the coating material for the phase modulation layer. Starting from the glass substrate, the structure is divided into four regions (A, B, C, and D) with different thicknesses but equal areas according to phase relationships, as shown in Table 2. The reflection phases of the four regions are 0°, 90°, 180°, and 270° for the 1.27 μm wavelength band. It should be noted that since the coating is applied on the exit surface of the glass substrate, which has a higher refractive index than air, this thin-film system inherently exhibits favorable wide-field characteristics. This configuration effectively reduces phase aberrations across different incident angles, ensuring consistent phase modulation performance over a broader angular range.

Layers 2 through 8 are composed of metal–dielectric reflective layers. Alternate deposition of high- and low-refractive-index materials is employed for layers 2 to 7, while layer 8 consists of silver. To streamline the coating process, the thicknesses of subsequent layers, starting from layer 2, are uniform across all four regions (A, B, C, and D), allowing for simultaneous deposition of the regions as an integrated structure. The high- and low-refractive-index materials are TiO_2_ and MgF_2_, respectively. The scheme for the dielectric film layers is shown in Table 3.

Layer 8 of the film is made of silver with a thickness of 0.05 mm, and all four zones are identical. Layer 9 is a protective layer that prevents the metal film from being exposed to air, thereby avoiding oxidation and wear. A protective Al_2_O_3_ coating material with a uniform thickness of 0.025 mm across all four zones is deposited simultaneously as an integrated structure.

### 3.3. Fabrication of the Stepped-Phase Mirror

This section describes the coating fabrication process for the designed stepped-phase mirror. The procedure is similar to the traditional front-side, four-quadrant stepped coating process, which has already been validated in an SWIMI experiment [17]. The coating is deposited using vacuum magnetron sputtering equipment, with high-temperature-resistant polyimide films employed as masks. Initially, the mask is affixed to the glass substrate and cut into four sections according to the designed dimensions, facilitating the easy removal of the mask from any specific area during the coating process.

**Figure 4:**
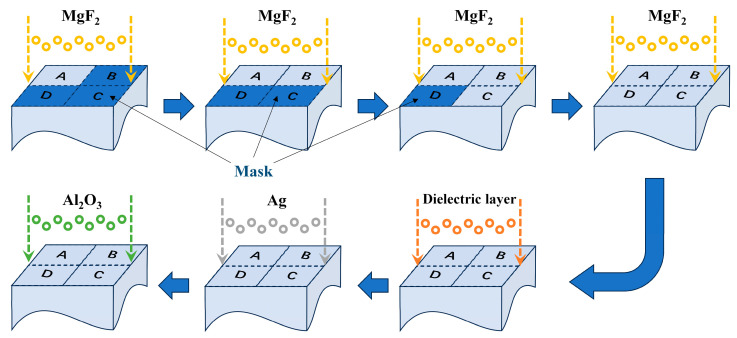
The fabrication process of the coating for the designed stepped-phase mirror.

As shown in Figure 4, fabrication starts with the deposition of the four-quadrant phase modulation layer using a cumulative coating approach. First, the mask from the region designed to have the thickest coating (e.g., region A) is removed, and a coating is applied. However, the deposited thickness at this stage is not the full designed thickness for region A but, rather, a thin layer whose thickness equals the difference between the designed thicknesses of region A and region B. Next, the mask from region B is removed, and coating continues—at this point, the thickness of region A increases further while region B begins to be coated, with both areas gaining the same additional thickness. This process is repeated for regions C and D in sequence, ultimately completing the deposition of the four-quadrant phase modulation layer. The specific coating steps and corresponding thicknesses are detailed in Table 4.

Subsequently, with all four-quadrant masks completely removed, the dielectric layer, Ag layer, and protected Al_2_O_3_ layer can be uniformly deposited, finally forming a complete coating system on the exit surface of the compensation glass.

The step-free coating technique proposed in this paper can simplify the assembly process of the four-quadrant wind imaging interferometer while significantly improving the stability of the instrument. In the traditional four-quadrant coating, since the coating is applied on the light incident surface, the four-quadrant, stepped-phase mirror is separated from the compensation glass. A gap must exist between them, and metal brackets are required for connection. During the assembly of the interferometer, it needs to be divided into two steps: first, install the compensation glass arm; then, install the four-quadrant, stepped-phase mirror. Such an assembly process is relatively complex and prone to introducing assembly errors multiple times.

**Figure 5:**
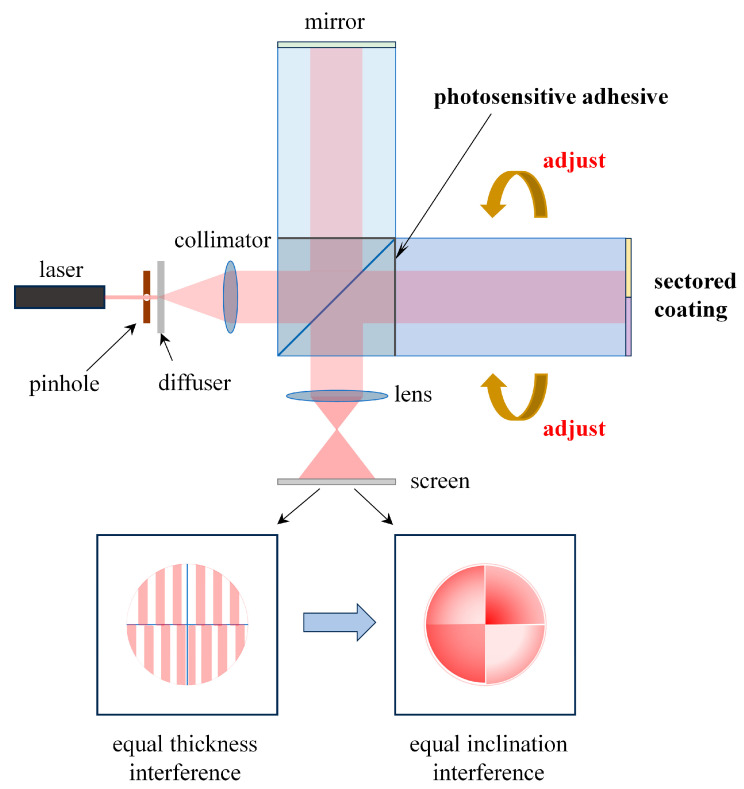
The assembly process of the compensation glass arm with the sectored coating.

The assembly process of the new scheme is shown in Figure 5. Since the four-quadrant, stepped-phase mirror is directly coated on the exit surface of the compensation glass, it is integrated with the compensation glass and does not require separate assembly. During the assembly process, first, apply photosensitive adhesive on the bonding surface of the beam splitter and the compensation glass. Then, place the entire system on a stable platform, use a helium–neon laser to build an optical monitoring system, and utilize a collimated laser beam to monitor the parallelism of the reflecting surfaces at the ends of the two arms of the interferometer in real time. By adjusting the orientation of the compensation glass arm, the interference pattern presented on the optical screen is transformed from equal-thickness interference fringes to equal-inclination interference fringes, as shown in Figure 5, which indicates that the parallelism of the reflecting surfaces of the two arms of the interferometer meets the requirements. Then, use an ultraviolet lamp to irradiate the glued surface for curing, and the assembly of the four-quadrant wind imaging interferometer is completed. This process can be formed in one step, which greatly simplifies the instrument assembly and adjustment process. Moreover, it does not rely on metal bracket connection; the improvement in its stability will be explained in Section 5.

## 4. Analysis

**Figure 6:**
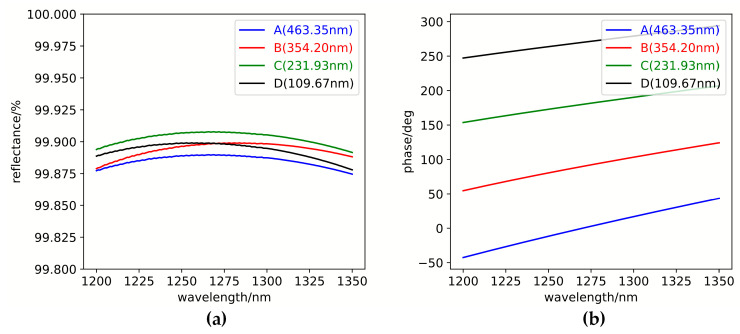
The variation of the reflectance and reflection phase across the four regions. (**a**) Wavelength-dependent variation of the reflectance. (**b**) Wavelength-dependent variation of the reflection phases.

The optical performance of the multilayer system, the including reflectance and reflection phase across all four zones, is analyzed and evaluated using optical thin-film design software. Since the design is based on a reference wavelength of 1.27 μm, both the reflectance and the step phase are optimized at this wavelength. Accordingly, it is necessary to analyze the reflectance and stepped-phase characteristics at both the three strong and three weak spectral lines. The reflectance and reflection phase as functions of wavelength are first analyzed over the range of 1200 nm to 1350 nm. The results are presented in Figure 6. As shown in Figure 6a, within the operational wavelength band of 1200–1350 nm, the designed four partitioned regions exhibit a reflectivity variation of approximately 0.035%, while all regions maintain reflectivity values exceeding 99.87%, with even higher reflectivity observed at the three strong lines and three weak lines. As illustrated by the phase characteristic analysis in Figure 6b, the reflection phases across the four zones demonstrate approximately linear wavelength dependence with gentle variations and consistent trends. The near-equal phase differences observed between distinct wavelengths substantiate that this thin-film system design effectively achieves controlled four-quadrant phase-stepping increments throughout the broad spectral range, with each partition exhibiting phase increments approaching 90°.

**Figure 7:**
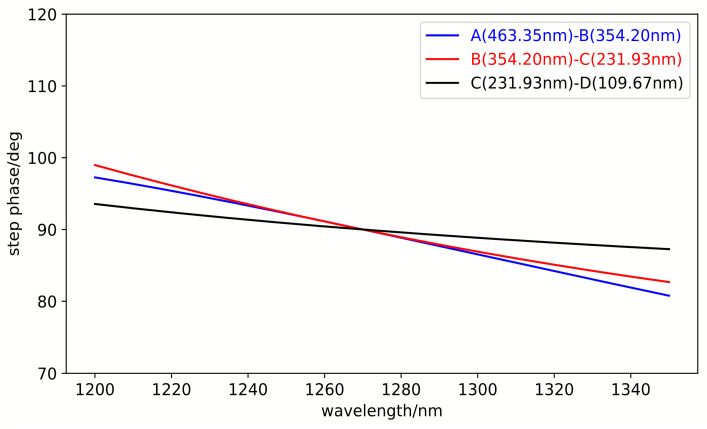
Wavelength-dependent variation of stepped reflection phases across adjacent regions.

Figure 7 illustrates the calculation of the reflective step-phase differences from region A to B, B to C, and C to D. As demonstrated, the stepped reflection-phase differences between adjacent regions remain fixed at 90° exclusively at the design wavelength of 1270 nm, whereas significant variations are observed across other wavelengths. However, the overall variation trend remains relatively gentle, with fluctuations within ±10° in the 1200–1350 nm wavelength range and within ±5° in the spectral regions corresponding to the three strong lines and three weak lines.

An alternative coating approach employs a multilayer structure consisting of a stepped-phase modulation layer combined with a metallic reflective layer. In this design, MgF_2_ with varying thicknesses is directly utilized as the stepped-phase modulation element, overlaid with a silver reflective film. The incident light enters regions A, B, C, and D; passes through the MgF_2_ layer with varying thicknesses; is reflected by the Ag layer; and exits after passing through the MgF_2_ layer again. Due to the differences in optical path lengths, this process results in different reflection phases. The absence of dielectric layers in this coating system simplifies the deposition procedure. The coating system was designed and analyzed using optical thin-film design software, with the MgF_2_ layer thicknesses for the four quadrants listed in Table 5.

**Figure 8:**
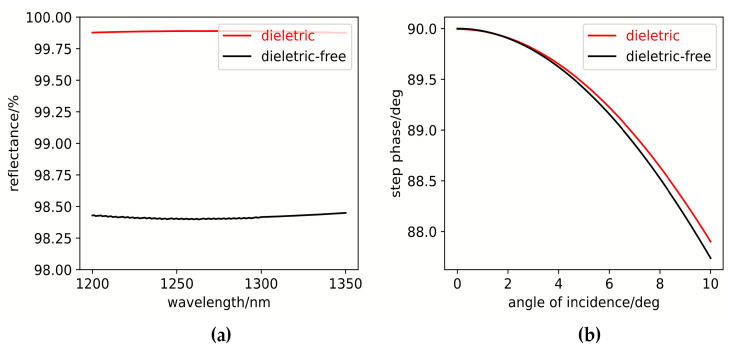
Comparation of the reflectance and step phase between the two coating architectures. (**a**) Variation of the reflectance. (**b**) Variation of the stepped reflection phase.

The film-system characteristics were analyzed using software and compared with the previously described four-quadrant, stepped-phase reflective coating containing dielectric layers. The results are shown in Figure 8. Figure 8a presents a comparison of the reflectance in region A as a function of wavelength for both film systems. The system incorporating dielectric layers achieves more than 1% higher reflectance than the dielectric-free system, which also displays considerable reflectance disparities among its four zones. These results underscore the improved performance afforded by the inclusion of dielectric layers. Figure 8b shows the variation of the stepped reflection phase from region B to C with incident angle, indicating that the dielectric-free coating is more sensitive to changes in the angle of incidence.

These results demonstrate that the novel film structure—comprising a phase modulation layer, a metal–dielectric reflective layer, and a protective layer—exhibits high reflectance and excellent uniformity across the four zones, thereby facilitating subsequent relative intensity calibration. This coating design furnishes the basic prerequisites for the development of wide field-of-view (FOV~10°) wind imaging interferometers. The system also requires fewer materials, incurs lower costs, presents reduced complexity in periodic deposition, and achieves high precision in stepped-phase control, thereby establishing a novel framework for all-solid-state compensation in static four-quadrant coated wind imaging interferometers.

To evaluate the manufacturing tolerance of the proposed coating design scheme, a Monte Carlo simulation was performed using the Sensitivity Analysis tool in TFCalc software. The thickness deviation of all film layers was set to 3%, and the refractive index deviation of the film materials was set to 2%, with a total of 1000 calculation analyses conducted. The quartile simulation results indicate that the second quartile (Q2) curve overlaps with the target curve of the coating design, which demonstrates that the performance meets the requirements under normal manufacturing errors. By calculating the Interquartile Range (IQR) based on the first quartile (Q1) and the third quartile (Q3), it is found that the fluctuation range of the reflectivity of the film system in each region is ±0.015% and the fluctuation range of the reflection phase is ±4°. These results satisfy the calibration requirements of the four-quadrant wind imaging interferometer [18]. Furthermore, due to the adoption of the coating method with mask coverage as shown in Figure 4, the step-phase errors between different regions will not accumulate. This ensures the accuracy of the step phase and further validates the reliability of the proposed coating design in practical manufacturing scenarios.

## 5. Discussion

This paper proposes a novel step-free coating technique that effectively mitigates optical path variation induced by surface roughness, thereby minimizing incident-light wavefront distortion. Compared with coating the front surface of the reflector’s glass substrate, coating the rear surface offers significantly higher flatness. Moreover, it is less prone to wear and tear and less susceptible to the adhesion of dust in the air, which can greatly enhance the stability of the thin-film system. This structural advantage stems from the fact that the rear surface, being less exposed to the external environment during operation, avoids direct contact with operational tools or external mechanical impacts, thereby reducing the risk of physical damage to the coating. Additionally, the relative isolation from air flow and particulate matter minimizes the accumulation of contaminants, which could otherwise alter the optical properties of the film over time. Such improved durability and environmental resistance are particularly critical for maintaining long-term phase modulation accuracy in wind imaging interferometers, where stable optical performance directly affects the precision of atmospheric wind field measurements.

**Figure 9:**
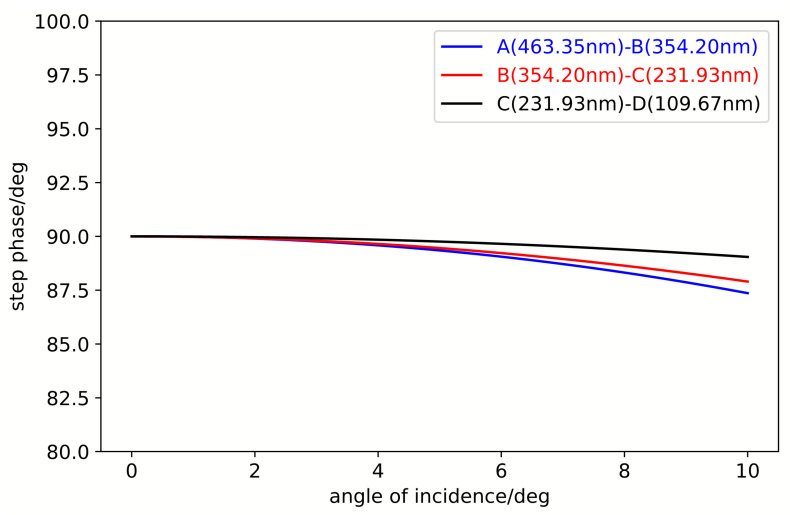
Dependence of the reflective stepping phase between adjacent partitions on the angle of light incidence.

The purpose of this design is to furnish a four-quadrant wind imaging interferometer with a solution that achieves both high reflectance and high accuracy in stepped-phase modulation. For the conventional static four-quadrant, air-gap wind imaging interferometer, the present design applies a coating to the rear side (light-exit surface) of the reflective mirror’s glass substrate. For the novel all-solid-state compensated wind imaging interferometer, the optical coating is directly deposited on the exit surface of the compensation glass substrate. By this approach, as mentioned above, performance is enhanced while both cost and manufacturing complexity are reduced. However, as evidenced by Figure 8b, the stepped-phase variation exhibits a strong dependence on incident angle for both metal–dielectric and dielectric-free coating architectures. Figure 9 demonstrates that the reflective stepping phase between adjacent segments varies as a function of the incident angle of light. Two key observations emerge: the absolute values of each stepped phase shift vary with incident angle, and the three phase steps exhibit distinct angular variation trends. However, the variation of the stepping phase with angle is minimal at an incident angle of 10°; the maximum change in the stepping phase does not exceed 3°. In wind imaging interferometers with semi-field angles typically below 5°, variations in adjacent-zone stepped phases induced by the incident angle are negligible in comparison with phase calibration errors.

**Figure 10:**
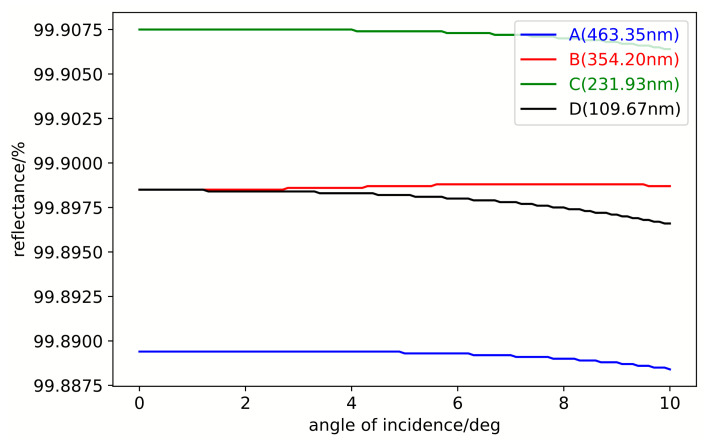
Incident-angle-dependent reflectivity characteristics in the step-free coating system.

In addition to wavelength, the reflectance of the film system also varies with the angle of incidence. An analysis was conducted for light with a wavelength of 1270 nm and an incident angle ranging from 0° to 10°. As shown in Figure 10, when the incident angle varies within the range of 0° to 10°, the reflectance in the four regions of the film system remains nearly unchanged, indicating that the reflectance is insensitive to the angle of incidence.

**Figure 11:**
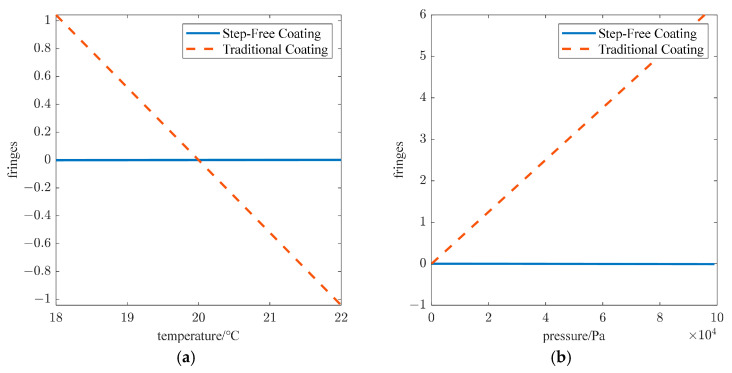
Drift of reference optical path difference between step-free coating and traditional coating with (**a**) temperature of the environment and (**b**) atmospheric pressure.

Next, we discuss the comparison of stability between the proposed method and existing technologies. In the traditional temperature compensation theory for wind imaging interferometers, only the thermal expansion effect of the compensation glass is taken into account. However, due to the presence of air gaps and limitations of coating technology, the reflectors in the two arms of the interferometer cannot be directly bonded to the compensation glass. Instead, additional mechanical structures are required for support. This issue significantly compromises the thermal stability of the wind imaging interferometer, and as such, stability becomes highly susceptible to the performance of the interferometer’s mechanical support system. Assume that the interferometer’s mechanical support is fabricated from aluminum. The coefficient of thermal expansion of aluminum is approximately 2.3 × 10^−5^/℃. Affected by the thermal expansion of the aluminum bracket, the absolute change rate of the reference optical path difference with temperature of the traditional coating in Figure 11a reaches 0.52 fringes/℃, which is far greater than the step-free coating design value of 3.27 × 10^−3^ fringes/℃. In practice, obvious four-zone interference intensity variation with temperature is observed in the SWIMI experiment, and additional correction for temperature drift is required.

On the other hand, the step-free coating scheme is far less susceptible to atmospheric pressure variations than the traditional coating scheme with an air gap. As atmospheric pressure changes, the refractive index of air is also altered, and this change induces a variation in the optical path of the air gap, ultimately leading to drift in the reference optical path difference. As shown in Figure 11b, the absolute change rate of the reference optical path difference with the atmospheric pressure of the step-free coating scheme is only 8.19 × 10^−5^ fringes/kPa, while the absolute change rate of the traditional coating scheme with an air gap reaches 6.28 × 10^−2^ fringes/kPa. The design of the new step-free coating scheme will greatly improve the environmental stability of the wind imaging interferometer and obtain higher detection accuracy of atmospheric dynamics parameters.

## 6. Conclusions

This design represents a novel coating technology that involves sequential deposition of a phase modulation layer, metal–dielectric reflective layer, and protective layer on the back side of compensation glass. The substrate’s surface flatness is optimized to minimize errors introduced by step discontinuities in the film stack. High- and low-reflectivity metal–dielectric layers are then alternately deposited to enhance overall optical reflectance. Finally, a protective overcoat is applied to prevent any reflectivity changes arising from reactions between the metal–dielectric layers and atmospheric oxygen. This technology achieves a step-free configuration in the four-quadrant, stepped-phase reflector, thereby providing a method with high reflectivity and high-precision stepped-phase control for wind measurement in four-quadrant wind imaging interferometers. The film system employs a minimal set of materials, thereby lowering costs and simplifying the deposition process. In this paper, we have presented a detailed description of a four-quadrant, step-free, stepped-phased reflective structure and the corresponding material selection, and then analyzed the sensitivity of the proposed method and verified its feasibility through simulations. The high-efficiency performance of this coating system lays a solid foundation for related technological research, such as in the field of all-solid-state compensation for wind imaging interferometers.

## Figures and Tables

**Table 1:** Environmental coating parameters.

Reference Wavelength (nm)	Incident Medium	Substrate	Exit Medium
1270.0	Glass	Ag	Air

**Table 2:** Thickness of phase modulation layers in the four-quadrant coating system.

Region	A	B	C	D
Thickness (nm)	463.35	354.20	231.93	109.67

**Table 3:** Dielectric coating architecture.

Layer Index	2	3	4	5	6	7
Material	TiO_2_	MgF_2_	TiO_2_	MgF_2_	TiO_2_	MgF_2_
Thickness (nm)	148.06	231.71	146.06	232.21	148.06	209.00

**Table 4:** The specific coating steps and corresponding thicknesses of the phase modulation layer.

Region	A	B	C	D
Step 1 (nm)	109.15	masked	masked	masked
Step 2 (nm)	122.27	122.27	masked	masked
Step 3 (nm)	122.26	122.26	122.26	masked
Step 4 (nm)	109.67	109.67	109.67	109.67
Total thickness (nm)	463.35	354.20	231.93	109.67

**Table 5:** Thickness of phase-modulation layers in dielectric-free optical coatings.

Region	A	B	C	D
Thickness (nm)	902.70	793.55	671.28	549.02

## Data Availability

The original contributions presented in this study are included in the article. Further inquiries can be directed to the corresponding author.
